# Impact of an intervention to support hearing and vision in dementia: The SENSE‐Cog Field Trial

**DOI:** 10.1002/gps.5231

**Published:** 2019-12-03

**Authors:** Iracema Leroi, Zoe Simkin, Emma Hooper, Lucas Wolski, Harvey Abrams, Christopher J. Armitage, Elizabeth Camacho, Anna Pavlina Charalambous, Fideline Collin, Fofi Constantinidou, Piers Dawes, Rachel Elliott, Sue Falkingham, Eric Frison, Mark Hann, Catherine Helmer, Ines Himmelsbach, Hannah Hussain, Sarah Marié, Susana Montecelo, Chryssoula Thodi, Wai Kent Yeung

**Affiliations:** ^1^ Global Brain Health Institute Trinity College Dublin Dublin Ireland; ^2^ Division of Neuroscience and Experimental Psychology University of Manchester Manchester UK; ^3^ Institute of Applied Research, Development and Further Education Catholic University of Applied Sciences Freiburg Freiburg Germany; ^4^ Department of Communication Sciences and Disorders University of South Florida Tampa FL; ^5^ Division of Psychology and Mental Health University of Manchester Manchester UK; ^6^ Manchester Centre for Health Economics University of Manchester Manchester UK; ^7^ Department of Health Sciences European University Cyprus Nicosia Cyprus; ^8^ INSERM, EUCLID/F‐CRIN Clinical Trials Platform University of Bordeaux Talence Aquitaine France; ^9^ Centre for Applied Neurosciences University of Cyprus Nicosia Cyprus; ^10^ Manchester Centre for Audiology and Deafness University of Manchester Manchester UK; ^11^ Department of Audiology Starkey Hearing Technologies Stockport UK; ^12^ Centre for Biostatistics University of Manchester Manchester UK; ^13^ INSERM, Bordeaux Population Health Research Center, Team LEHA University of Bordeaux Talence Aquitaine France; ^14^ Research and Development Essilor International Paris France

**Keywords:** complex intervention, dementia, hearing impairment, quality of life, resource utilisation, vision impairment

## Abstract

**Objectives:**

Hearing, vision, and cognitive impairment commonly co‐occur in older adults. Improving sensory function may positively impact outcomes in people with dementia (PwD). We developed a “sensory intervention” (SI) to support hearing and vision in PwD. Here, we report the findings of an international open‐label field trial, and nested case series, to explore the impact of the SI on dementia‐related outcomes.

**Methods:**

This was a home‐based trial conducted in France, England, and Cyprus. Participants were people with mild‐to‐moderate dementia and hearing and/or vision impairment (n = 19) and their study partners (unpaid carers; n = 19). The “basic” SI included a hearing and vision assessment and provision of glasses and/or hearing aids. A subsample received the “extended” SI with additional weekly visits from a sensory support therapist (SST). Exploratory analyses of dementia‐related, health utility and resource utilisation outcomes were performed.

**Results:**

Quality of life (QoL) and sensory functional ability improved. Change in QoL exceeded the threshold for a minimum clinically important difference. There was a modest improvement (in absolute terms) post intervention in behavioural disturbance, self‐efficacy, and relationship satisfaction. Study partner time assisting instrumental activities of daily living (iADL) and supervision decreased by about 22 and 38 hours per month, respectively, although time for personal ADL support increased. Qualitative data supported effectiveness of the intervention: PwD were more socially engaged, less isolated, less dependent on study partners, and had improved functional ability and communication.

**Conclusions:**

These findings support the need for a definitive randomised controlled trial (RCT) to evaluate the effectiveness of the intervention.

**Key points**

- Hearing, vision, and cognitive impairment commonly co‐occur in older adults and improvement in sensory function may positively impact outcomes in people with dementia (PwD).
- A sensory intervention (SI), involving both hearing and vision support, was developed for PwD, with a view to improve quality of life.
- Findings support the need for a full‐scale randomised controlled trial to evaluate the effectiveness of hearing and vision support in PwD and their care partners.

## 1 INTRODUCTION

Hearing and vision impairments are more common in people with dementia (PwD) than in those who are cognitively healthy.^1^, ^2^ Such impairments negatively affect a range of dementia‐related outcomes, including quality of life (QoL), behaviour, and cognition, as well as imposing an added burden on health, social, and informal care.^3^, ^4^, ^5^, ^6^ There is some evidence that correcting vision and hearing impairments with glasses and hearing aids, respectively, may improve outcomes,^7^ but in PwD, adherence is often low.^8^ Thus, correcting the sensory impairment alone may be insufficient and multifaceted interventions, including adherence support and communication training, may be needed. To address this, we developed a “sensory intervention” (SI) comprising assessment and management of sensory deficits, support with adherence and maintenance of devices, communication training, sensory enhancement of the home environment, and signposting to additional support services.^5^

The SI was developed, using an intervention mapping process, in alignment with the theoretical framework of the evidence‐based model of the Behavioural Change Wheel, particularly, the COM‐B component.^9^ According to this model, key areas to be addressed to effect behavioural change (“B”) include *capability* (“C”) or the individual's psychological and physical capacity to engage in the activity concerned; *opportunity* (“O”) or all the factors that lie outside the individual that make the living better with dementia possible; and *motivation* (“M”) or the brain processes that direct goals and decision making. Each component of the SI corresponded with one or more of the COM‐B elements, as outlined in detail previously.^5^

A handful of interventions aimed at improving hearing or vision impairment already exist, however, they either do not address the problem of *combined* hearing and vision impairment or are not aimed at PwD.^7^ Importantly, to be effective, interventions for sensory remediation should address the specific needs of each individual, arguing for highly tailored interventions rather than “off the shelf” or more generic approaches.^2^, ^6^, ^10^

The SENSE‐Cog Field Trial was a key step in the iterative development of the SI, and followed the guidance from the UK's Medical Research Council on the development of complex interventions.^11^ Our aim was to inform the design and conduct of a subsequent full‐scale randomised controlled trial (RCT) to evaluate the effectiveness of SI on dementia‐related outcomes. Our field trial had two objectives (a) to evaluate the feasibility, acceptability, and tolerability of the study procedures and new intervention (reported separately)^12^ and (b) to explore a signal of effectiveness and identify key drivers of resource use (for a cost‐effectiveness evaluation) of this type of intervention (reported here). The full‐scale RCT opened to recruitment in Spring 2018^5^, ^13^ in five European sites: Athens, Dublin, Manchester, Nice, and Nicosia.

## 2 METHODS

### 2.1 Study design and participants

This was a single‐arm, open‐label field study including 19 dyads (PwD and their study partner). Study partners were family members or close friends who knew the PwD well and had a role as an unpaid carer, supporting the PwD with activities of daily living. They were coresident or in regular contact (at least twice per week) with the PwD. Study sites were Bordeaux, France (site B), Manchester, United Kingdom (site M), and Nicosia, Cyprus (site N). All participant dyads received the *basic* form of the SI. A subset of four dyads in site M received an *extended* version, a multipart intervention comprising the basic SI *plus* a package of support from a “sensory support therapist” (SST). The SST delivered the intervention to both the PwD and their study partner. We conducted semistructured interviews with a subsample at sites M and N who received either the basic (n = 8) or extended (n = 2) SI. We also include a nested case series of a purposive sample of four dyads who received the extended SI.

We recruited participants from memory assessment clinics or through dementia research registries. Inclusion and exclusion criteria are outlined in Table 1 and included older adults with a formal diagnosis of a common form of dementia (Alzheimer disease, vascular dementia, or mixed dementia) who also had adult‐acquired mild‐to‐moderate hearing and/or vison impairment (see Table 1 for detailed criteria for “impairment”). We excluded those with unstable medical or psychiatric conditions or congenital sensory impairments. The study received favourable ethical opinion at all sites, as per local requirements.

**Table 1:** Summary of participant inclusion and exclusion criteria and characteristics

Participant Study Criteria Detailed previously in Reganet al^14^		
Inclusion	Age	≥60 y
	Domiciliary status	Community based (living at home)
	Cognitive impairment	Formal clinical diagnosis of a common form of dementia (Alzheimer disease, vascular dementia or “mixed” Alzheimer, and vascular dementia)^a^
	Stage of cognitive function	Early‐moderate stage dementia (as per the MoCA score of ≥12)
	Hearing or vision impairment or both	Screening positive for hearing impairment defined as bilateral hearing difficulty (ie, failure on a pure tone hearing screening test (using the handheld HearCheck screening device^15^ in both ears—hearing >35 dB HL over 1‐3 kHz and above in the better ear; the HearCheck screener provides a count of detected signals at or above threshold levels for two frequencies (three levels per frequency) and gives the total number of tones detected from 0‐6 for each ear. A participant was considered to have a “positive” screen with any score less than 6 in both ears.
		Screening positive for vision impairment defined as distance binocular visual acuity with current equipment ≤6/9.5 and >6/60 in Snellen metric (or ≥+0.2 logMAR [75 EDTRS Score] and <+1.0 logMAR [35 EDTRS Score]) and visual field >10°; screened using the PEEK tool^16^ a smartphone‐based visual acuity check app alongside the confrontation visual field test: “can you see my hands?.”
	Capacity to consent to the study	Assessed using the criteria for capacity defined by the UK's Mental Capacity Act (2005)^17^
	Study partner	An adult study partner familiar with and in regular contact with (at least twice a week) the participant with dementia and their needs and supporting their activities of daily living
Exclusion criteria	Hearing or vision impairment	Congenital hearing and/or vision impairments
	General status	Any unstable, acute physical or mental condition that would preclude participation in the study

Abbreviation: MoCA, Montreal Cognitive Assessment.^18^

a Diagnosed with dementia in accordance with ICD10 (10th revision of the International Statistical Classification of Diseases and Related Health Problems) criteria because of the following conditions: Alzheimer disease (in accordance with NINCDS‐ADRDA^19^ criteria) or vascular dementia (in accordance with NINDS‐AIREN^20^ criteria) or “mixed” dementia.

We have detailed participant characteristics in Table 2. Briefly, PwD were above age 62 years, and study partners were above age 42. Of the PwD, 42% (n = 8) had hearing impairment only; 58% (n = 11) had both vision and hearing impairment; and none had vision impairment alone. There was an equal proportion of PwD due to Alzheimer disease and vascular dementia; and one individual had “mixed” dementia.

**Table 2:** Summary of participant demographics and clinical characteristics

Participant Demographic and Clinical Characteristics at Baseline (n = 19 dyads)		
PwD	Age	Median 76 y (IQR, 11; range 63‐88)
	Male sex	63% (n = 12)
	MoCA	Mean 17.3 (SD 3.7; range 12‐23)
	Duration of cognitive impairment	Median 60 mo (IQR, 54; range 6‐120)
	Dementia type	Alzheimer disease (n = 9); vascular dementia (n = 9); mixed dementia (n = 1)
	Hearing impairment only	42% (n = 8)
	Vision impairment only	None
	Combined hearing and vision impairment	58% (n = 11)
	Level of hearing impairment in those screened positive	n = 12 detected the same number of tones in both ears out of 6 (2 tones = 1, 3 tones = 4, 4 tones = 5, 5 tones = 2); n = 7 had scores of 3/2, 1/3, 1/3, 2/5, 3/5, 4/5, 4/5 for left and right ears, respectively
	Level of visual impairment in those screened positive	n = 9 had mild impairment (+0.2 to 0.5 LogMAR); n = 2 had moderate impairment (+0.6 to 0.9 LogMAR) (ICD10 classification^21^)
Study partners	Age	Median 67 y (IQR, 13; range 43‐82)
	Sex	68% (n = 13)
	Coresident with participant with dementia	79% (n = 15)

Abbreviations: IQR, interquartile range; MoCA, Montreal Cognitive Assessment.^18^

### 2.2 Description of the intervention

The *basic* SI involved a detailed vision and/or hearing assessment (home or clinic based), followed by prescription and fitting of lenses or hearing aids, as indicated by the assessment. The sensory assessments were carried out by audiologists and/or optometrists in a manner suitable to people with cognitive impairment, including allowing more time for the assessment, clear explanations of each step and written information regarding the procedures and devices prescribed. Hearing aids were “behind the ear” Muse Mini BTE i2400 (Starkey Hearing Technologies), and lenses were provided by Essilor International according to individual visual needs. This was followed by basic advice in using and maintaining the sensory devices that did not extend beyond the fitting visit. The *extended* SI (fully described in Regan et al^14^) comprised additional components: (a) individualised adherence support, comprising repeated visits for detailed assessment and advice regarding the use and maintenance of the devices; (b) communication training with the study partner; (c) referral to health and social care services to address comorbid problems; (d) supplementary aids to enhance sensory function in the home environment (ie, glasses straps, hearing aid clips, special lighting, and ambient noise management); and (e) support for social inclusion through hobbies, interest, and social groups.

### 2.3 Study procedures

The study protocol is detailed in‐depth elsewhere and is also outlined in a flow diagram of study procedures in Chart S1.^14^ Briefly, after informed consent, we screened PwD for sensory and cognitive impairment, followed by a baseline assessment of feasibility and outcome measures. We then delivered the basic SI for up to four visits (over 4 weeks) at all three sites. At site M, we offered the extended SI for up to 12 visits (over 8 weeks) to a purposive subset of participants with different combinations of hearing and/or vision impairments. The basic SI offered to all dyads was to ascertain key feasibility aspects of the intervention and to assess the dyads kept diaries of each visit, and the SST kept a log book detailing visits and dyad responses. Outcomes were assessed 1 week after the final intervention visit, and in a purposive subset of 10 dyads (five dyads at each of sites M and N) semistructured interviews were conducted to explore how dyads received the intervention and perceived its effectiveness. The details of the qualitative methods applied, including the use and development the topic guide for the interviews, is outlined in detail in the published protocol.^13^, ^14^

### 2.4 Battery of effectiveness measures

As outlined in Table S2, for the PwD, we assessed cognition, behaviour, mental well‐being, and self‐efficacy. For the study partner, we included measures of physical and mental well‐being and caregiver burden. We assessed relationship satisfaction and QoL in both dyad members. To calculate the health status of the PwD for the economic analysis, we collected generic (EQ‐5D‐5L)^22^ and dementia‐specific (DEM‐QoL)^23^ measures (self‐rated and proxy rated),^24^ and the Resource Utilisation in Dementia (RUD) Lite,^25^ to capture resource use of the PwD (during the month prior to baseline, and at follow‐up, since baseline).

In those who received the extended SI, we explored additional outcomes using (a) *goal setting* and (b) *participant diaries* and *SST logbooks*.

*Goal setting*: Goal setting in this study was used as a flexible, yet validated method of capturing meaningful, robust outcomes. A maximum of three goals were set relating to components of the intervention, using the Bangor Goal Setting Inventory (BGSI).^26^ Following goal identification, the SST explored facilitators and barriers to goal attainment and supported progress through introducing skills and strategies. The PwD and study partner individually rated performance on introduction of the goal and at review on a 10‐point scale (1 = “cannot do successfully” to 10 = “can do successfully”). The SST rated goal attainment at review on a 5‐point scale (0%, 25%, 50%, 75%, or 100%).

*Participant diaries and SST logbooks*: These included free‐text spaces for feedback, and Likert‐style ratings of the SI, on (a) level of engagement of the PwD (study partner and SST rated: 1 = strongly disagree to 5 = strongly agree) and (b) perceived helpfulness (dyad rated: 1 = “not at all helpful” to 5 = “very helpful”). The SST logbook also included measures of the PwD's hearing aid use skill and ability (Hearing Aid Skills and Knowledge Questionnaire; HASK)^27^ and glasses use skill and ability (a bespoke Glasses/Vision Skills and Knowledge Test).

## 3 DATA ANALYSIS

### 3.1 Quantitative analysis

As an initial exploration of a novel intervention, our goal was to observe any signal of change across various outcome measures. We examined the change between baseline (preintervention) and follow‐up (post intervention) by summarising distributions of outcome measures with measures of central tendency (mean or median) and variability (SD or interquartile range [IQR]). The small sample size precluded investigation of associations among outcomes. The covariates of interest were not heavily skewed, and mean and medians were similar; thus, we report mean values here.

### 3.2 Economic analysis

Our aim here was to test the performance of tools to measure health status and the use of health and social care resources including informal care in preparation for the economic evaluation of the full SENSE‐Cog RCT. In the full RCT, we will quantify the incremental changes in costs and benefits between the intervention and care as usual, from the health care provider and societal perspectives. Here, our measure of benefit for the primary analysis was health utility, derived from the EQ‐5D‐5L and value set for England.^28^ Secondary analyses derived utility values from the DEM‐QoL and respective value set for England.^29^ We also summarised utility values based on self‐rated and proxy‐rated responses on both questionnaires and described the use of health and social care resources using the RUD‐Lite questionnaire.^25^

### 3.3 Qualitative analysis

The free‐text feedback of the participant diaries and SST logbooks, as well as the postintervention semistructured interviews, were evaluated according to summative content analysis methodology, a reliable method of analysing of qualitative data using “coding units.”^30^, ^31^

## 4 RESULTS

Of the 19 participant dyads, eight each were from site M and site N, and three were from site B. Site B did not complete outcome assessments (detailed in Hooper et al^12^).

### 4.1 Effectiveness outcomes

Descriptive statistics of outcome variables are presented in Table 3. The average difference on the DEM‐QoL,^23^ the main outcome for the trial, post intervention was 4.87 points (a 4‐point improvement is considered clinically important), indicating that PwD felt their QoL had improved following the SI. All eight PwD from site M showed improvement (between 3 and 28 points), whereas participant responses from site N varied (two worsened, two no change, four improved; range −15 to 8 points). The DEM‐QoL‐Proxy,^39^ rated by the study partner on behalf of the PwD, showed an average decline of 2.53 points, with 10 of the 15 study partners reporting a decline post intervention, although this did not fall above threshold for a minimum clinically important difference, which may be as high as 6 points.^40^ In PwD, both hearing and vision functional ability improved post intervention, as reflected by lower scores on the HHIE‐S^34^ and higher scores on the LV‐VFQ‐20^35^ visual ability subscale.

**Table 3:** Baseline and postintervention measurements for the individuals with dementia and hearing and/or vision impairment (n = 19 dyads)

Outcome Domain^a^	Mean (SD); Range			
	Baseline^c(^Full Sample)	Baseline^c^ (Completers)	Post Intervention^c^	Difference
DEM‐QoL	93.42 (10.92)	92.27 (11.14)	97.13 (9.69)	4.87 (9.93)
	61 to 106	61 to 106	77 to 110	−15 to 28
DEM‐QoL‐Proxy	100.94 (11.49)	101.73 (11.45)	99.20 (11.85)	−2.53 (6.45)
	85 to 121	85 to 121	79 to 120	−13 to 8
HHIE‐S (only hearing impairment)	8.52 (8.08)	7.87 (7.95)	6.53 (3.96)	−1.33 (6.53)
	0 to 26	0 to 26	0 to 16	−10 to 8
LV‐VFQ‐20 (only vision impairment)	2.54 (1.14)	2.65 (1.13)	3.45 (1.00)	0.80 (1.16)
	0.86 to 4.22	0.86 to 4.22	1.59 to 4.79	−0.23 to 3.68
SF‐12 PCS	45.74 (11.85)	44.21 (12.48)	45.06 (11.01)	0.86 (6.47)
	23.36 to 56.82	23.36 to 56.23	22.96 to 57.23	−13.12 to 10.65
SF‐12 MCS	49.38 (10.10)	50.02 (9.73)	50.88 (11.22)	0.86 (9.65)
	35.12 to 60.70	35.12 to 60.70	22.12 to 68.41	−13.00 to 22.19
SF‐12 Proxy PCS	42.02 (10.46)	42.24 (10.44)	45.12 (10.21)	2.87 (9.19)
	21.23 to 55.50	21.23 to 55.50	24.75 to 57.82	−12.90 to 18.2
SF‐12 Proxy MCS	49.27 (10.45)	51.12 (8.60)	50.44 (7.18)	−0.68 (7.49)
	26.81 to 62.28	35.38 to 62.28	37.81 to 58.19	−15.43 to 11.74
NPI‐12^b^	13.74 (16.72)	9.80 (9.10)	8.60 (7.96)	−1.20 (11.42)
	0 to 66	0 to 32	0 to 26	−28 to 20
GSE	2.85 (0.54)	2.76 (0.50)	2.89 (0.56)	0.13 (0.46)
	2.1 to 3.9	2.1 to 3.7	2.1 to 4.0	−0.5 to 1.1
BADLS	12.50 (8.21)	11.73 (7.99)	14.33 (7.93)	2.60 (3.33)
	1 to 31	1 to 31	0 to 34	−5 to 8
RSS	27.21 (6.81)	29.13 (1.77)	29.60 (0.74)	0.47 (1.64)
	2 to 30	25 to 30	28 to 30	−2 to 5

Abbreviations: BADLS, Bristol Activities of Daily Living Scale^32^; DEM‐QoL(‐P), dementia quality of life (‐Proxy)^23^, ^29^; GSE, Generalised Self‐Efficacy^33^; HHIE‐S, Hearing Handicap Inventory for the Elderly Screening tool^34^; LV‐VFQ‐20, Low Vision Visual Functioning Questionnaire‐20^35^; MCS, mental component score; NPI‐12, Neuropsychiatric Inventory 12^36^; PCS, physical component score; RSS, Relationship Satisfaction Scale^37^; SF‐12, 12 Item Short Form Survey.^38^

a Four participant dyads completed the baseline evaluations only, thus no postintervention data were obtained on them. One dyad within the extended intervention group withdrew due to the burden of study visits, and the remaining three dyads were lost to follow‐up due to lack of feasibility of the site to deliver the intervention.

b Magnitude score = frequency × severity.

n = 18 or 19, except for LV‐VFQ‐20 (n = 11).

c n = 15, except for LV‐VFQ‐20 (n = 10) and GSE (n = 14).

There was a modest improvement, in absolute terms, post intervention, on other outcomes for the PwD. There were minimal changes on the PwD self‐rated physical and mental functioning component scores and on the proxy‐rated mental component score but a small increase in the PwD proxy‐rated physical functioning score post intervention. Slight improvements were observed in informant‐rated neuropsychiatric symptoms (on the NPI‐12)^36^ and general self‐efficacy (on the GSE scale).^33^ Relationship satisfaction (RSS)^37^ at baseline was rated as high by both PwD and study partners at baseline. For the PwD, this improved following the intervention, but for the study partner, this decreased slightly. Finally, functional ability in the PwD, ascertained using the BADLS,^32^ decreased slightly following the intervention (a 2.6‐point average score increase); however, this change did not reach threshold for a minimum clinically important difference (at least a 3.5‐point difference.)^41^

In study partners, as presented in Table 4, mean self‐rated depression using the GDS‐15^45^ reflected a low baseline level of depression (<5), with minimal change post intervention. Caregiving, rated using three subscales of the FCRS,^42^ showed minimal changes in levels of caregiving‐related satisfaction, resentment, and anger from baseline to post intervention. Finally, physical well‐being, rated using the 15‐item PHQ‐15,^44^ ranged from 1 to 15 (on a 30‐point scale) at baseline, and there was a small deterioration (an average of 1.1 points) post intervention.

**Table 4:** Baseline and postintervention measurements for the study partner of individuals with dementia (n = 19 dyads)

Outcome Domain^a^	Mean (SD); Range			
	Baseline^b^ (Full Sample)	Baseline^c^ (Completers)	Post Intervention^c^	Difference
GDS‐15	3.47 (2.87)	3.08 (2.75)	3.62 (2.66)	0.54 (2.44)
	0 to 8	0 to 8	0 to 10	−3 to 4
FCRS Satisfaction subscale	26.28 (5.69)	27.20 (5.63)	28.00 (3.51)	0.80 (4.06)
	12 to 33	12 to 33	21 to 32	−4 to 14
FCRS Resentment subscale	13.24 (4.44)	13.53 (4.26)	13.87 (4.34)	0.33 (3.64)
	5 to 20	5 to 20	6 to 22	−8 to 9
FCRS Anger subscale	7.83 (2.79)	7.53 (2.13)	7.93 (2.89)	0.40 (2.10)
	4 to 15	4 to 12	4 to 13	−3 to 4
PHQ‐15	6.18 (4.52)	6.15 (3.85)	7.34 (5.08)	1.19 (3.59)
	0 to 16	0 to 15	1 to 19.2	−3 to 11.2
RSS	23.63 (9.93)	27.36 (2.71)	26.64 (3.23)	−0.71 (2.67)
	1 to 30	21 to 30	18 to 30	−7 to 3

Abbreviations: FCRS, Family Care Giving Role Scale^42^; GDS‐15, Geriatric Depression Scale 15^43^; PHQ‐15, Patient Health Questionnaire 15‐Items^44^; RSS, Relationship Satisfaction Scale.^37^

a Four participant dyads completed the baseline evaluations only, thus no postintervention data were obtained on them. One dyad within the extended intervention group withdrew due to the burden of study visits, and the remaining three dyads were lost to follow‐up due to lack of feasibility of the site to deliver the intervention.

b n = 18, except for GDS‐15 and FCRS Resentment (n = 17) and RSS (n = 19).

c n = 15, except for GDS‐15 (n = 13) and RSS (n = 14).

### 4.2 Health economic outcomes

Resource use and health utility are summarised in Table 5. Study partners' average time spent assisting with personal activities of daily living (ADL) increased by 17 h/mo; however, time spent assisting with instrumental ADL decreased by 22 h/mo, and time spent supervising declined by 39 h/mo. Proxy‐rated health utility was lower than self‐rated utility according to both instruments (EQ‐5D‐5L and DEM‐QoL). In contrast, self‐rated utility on the DEM‐QoL increased, whereas proxy‐rated utility decreased.

**Table 5:** Baseline and postintervention outcomes for economic evaluation (n = 19 dyads)

Outcome Domain^a^	Mean (SD); Range			
	Baseline^b^ (Full Sample)	Baseline^c^ (Completers)	Post Intervention^c^	Difference
Resource Utilisation in Dementia (RUD) Lite^25^ (at baseline: resource use in previous month; at follow‐up: resource use since baseline)				
Number of caregivers in addition to primary caregiver	1.1 (1.2)	1.1 (1.3)	0.8 (1.2)	−0.3 (1.0)
	0 to 4	0 to 4	0 to 4	−2 to 2
Caregivers in paid employment	7/19 (37%)	5/14 (36%)	5/14 (36%)	0
Informal care time				
Time spent assisting with PADL,^d^ h/mo	29.6 (48.8)	37.5 (1.3)	54.2 (94.4)	16.7 (57.1)
	0 to 3	0 to 145	0 to 300	−60 to 155
Time spent assisting with IADL,^d^ h/mo	70.7 (79.3)	79.5 (90.4)	57.5 (52.5)	−22.0 (97.7)
	0 to 300	0 to 300	1 to 150	−295 to 90
Time spent supervising,^d^ h/mo	61.9 (111.6)	81.9 (124.7)	43.2 (75.2)	−38.7 (86.3)
		0 to 360	0 to 210	−200 to 110
	0 to 360			
Number of consultations with doctor, psychologist, physiotherapist, or other HCP	1.4 (1.2)	1.5 (1.1)	1.5 (1.6)	0.1 (1.6)
	0 to 4	0 to 4	0 to 5	−3 to 3
Use of services, home help, district nurse visits, day care, or other social care services	3.2 (6.3)	3.5 (6.9)	3.0 (6.3)	−0.5 (6.5)
	0 to 20	0 to 20	0 to 20	−20 to 12
PwD's health utility, instrument derived from (EQ‐5D‐5L or DEM‐QoL) and assessor (self‐rated or proxy rated)				
EQ‐5D‐5L generated utility (self‐rated)	0.83 (0.17)	0.82 (0.18)	0.82 (0.14)	0.00 (0.12)
		0.5 to 1	0.6 to 1	−0.2 to 0.3
	0.5 to 1			
EQ‐5D‐5L generated utility (proxy rated)	0.77 (0.21)	0.80 (0.17)	0.82 (0.15)	0.03 (0.08)
		0.5 to 1	0.6 to 1	‐0.2 to 0.2
	0.2 to 1			
DEM‐QoL generated utility (self‐rated)	0.87 (0.10)	0.87 (0.11)	0.92 (0.07)	0.05 (0.10)
		0.6 to 1	0.8 to 1	−0.1 to 0.3
	0.6 to 1			
DEM‐QoL generated utility (proxy rated)	0.82 (0.11)	0.81 (0.12)	0.79 (0.12)	−0.02 (0.1)
		0.5 to 0.9	0.5 to 0.9	−0.2 to 0.1
	0.5 to 0.9			

Abbreviations: DEM‐QoL, dementia quality of life^23^, ^29^; EQ‐5D‐5L, EuroQol Five Dimensions Five Levels^28^; HCP, health care professional; iADL, instrumental activities of daily life; PADL, personal activities of daily life.

a Four participant dyads completed the baseline evaluations only, thus no postintervention data were obtained on them. One dyad within the extended intervention group withdrew due to the burden of study visits, and the remaining three dyads were lost to follow‐up due to lack of feasibility of the site to deliver the intervention;

b n = 19, except for DEM‐QoL generated utility proxy (n = 18).

c n = 14/15, except for EQ‐5D‐5L generated utility proxy (n = 16).

d Hours/month derived from hours/day and days/month.

### 4.3 Qualitative findings from semistructured interviews

In Table S3, we have summarised the key themes from the semistructured interviews, illustrated by exemplar quotes from participants. These revealed positive effects of the intervention, including applicability of the SI to everyday life. Key themes to emerge included an improvement in *communication skills* within the dyad. For both members of the dyad, *knowledge*, *awareness*, and *understanding* of the nature and cause of the cognitive, behavioural, and functional changes in the PwD improved, as well as the use and maintenance of the devices. In a few cases, PwD took over the management of their hearing aids independently. Another theme was the *advantages of a home‐based intervention* with therapist support. This approach made participants feel comfortable, enhanced adherence to using the hearing aids and glasses, and fostered the view of the therapist as a “friend.” These themes were supported and extended by the free‐text comments in the participant diaries (Table S4).

### 4.4 Nested case series for the extended intervention recipients

In Table S4, we describe in detail the four dyads who received the extended SI.

#### 4.4.1 Description of cases and findings from the extended SI

Three PwD completed the full intervention (cases 1 to 3; all received hearing aids and one received glasses). One PwD (case 4) withdrew after four visits due to anxiety, although he accepted hearing aids and glasses, after which his study partner reported that his communication improved. In the completing dyads, hearing aid skills and knowledge improved with SST support. Adherence to hearing aids was as prescribed (ie, waking hours) in three of the four PwD who received the aids. The nonadherent participant (case 3) had problems with the fit of the hearing aid and his use diminished over time.

#### 4.4.2 Goal setting outcomes

Dyads set between two to three goals around the following themes: device use, device care, communication, function, and social inclusions. Mean goal changes post intervention were all positive and ranged from 2.7 to 4.2 points for PwD and 4.3 to 4.7 points for study partners, on the 10‐point scale. Of the nine completed goals, nearly all (89%) were rated at least 75% attained by the SST, which we considered acceptable.

#### 4.4.3 Participant diary and therapist logbooks

PwD mean diary ratings of “helpfulness” of the SI components indicated moderately high (≥3 out of 5) “helpfulness.” Study partner diary and SST log book mean ratings of PwD “initiative” both indicated a range from “disagree” to “strongly agree.” Qualitative findings from the dyad diaries and SST logbooks reported improved hearing function with an overall positive impact of the SI, supporting the findings from the semistructured interviews. For example, dyads reported notable improvements in the PwD's performance of ADL and resumption of previous activities, which they felt related to improved hearing. This, in turn, lessened their dependence on their study partner through increased confidence and independence. Study partners reported that receiving the intervention led to improved communication and relationship quality for the dyad, and the PwD being able to follow multiple strands of conversation and being more engaged with family life. One study partner reported, “I feel like I've got him back.” PwD themselves reported feeling “more confident” and “vocally alive.” Participant dyads also described how hearing aids helped combat the social isolation due to the combined cognitive‐sensory impairments, and fostered a positive affect, which impacted positively on their relationship, supporting the quantitative findings.

## 5 DISCUSSION

This is the second report of the SENSE‐Cog Field Trial detailing the impact of a home‐based intervention of both hearing and/or vision remediation in people living with dementia and their study partners. In the first report, we detailed the feasibility of conducting such a study in this population, as well as the acceptability and tolerability of the intervention itself.^12^ Findings in that report have informed the design and conduct or a larger, follow‐on RCT.^13^ Here, our exploratory analysis has revealed that managing hearing and vision impairment in PwD has a positive impact on a number of outcomes, including QoL, self‐efficacy, and neuropsychiatric symptoms. For those receiving the extended intervention with SST input, synthesis of qualitative, diary and SST logbook findings clearly supported the intervention: PwD were more socially engaged and less isolated and reported improvements in functional ability and communication. They were less dependent on study partners. Given the limitations of the sample size, it appears that there is a good range of scores across all outcomes. There was a mix of sizeable positive changes (mostly PwD), and some small negative changes (mostly study partners), in individual scores post intervention.

Our finding of a positive impact on health‐related QoL (HRQL) in PwD is important; we have thus selected this as the primary outcome for the follow‐on RCT, using the DEM‐QoL. Mean baseline DEM‐QoL ratings of our study sample were similar to other studies of psychosocial interventions for PwD,^46^, ^47^ and the improvement noted was reflected not only in the DEM‐QoL score but also in health utility values derived from the DEM‐QoL, and the positive qualitative feedback on the intervention by both members of the dyads. Differences in the DEM‐QoL change scores between sites may be due to cultural factors, which will be explored in a detailed process analysis in the follow‐on RCT. The specific positive findings on social isolation, resumption of previous activities, and self‐efficacy rating further underscore the favourable impact of the intervention on HRQL.

The improvement in functional ability found in the qualitative data was interesting, considering BADLS ratings revealed a slight worsening of postintervention scores. However, we have interpreted the quantitative outcomes in our study with caution since it was not designed to evaluate effectiveness, and rating scale outcomes were intended only to provide a proof of concept of the intervention to inform a fully powered RCT. Importantly, the drop in number of caregiving and supervision hours per month reinforced our finding of an improvement in self‐efficacy in the PwD. The increase in personal care provided may reflect the additional time spent supporting the PwD's use of the sensory aids. Considering that informal care for PwD has been valued at nearly £132 billion per year,^48^ improving sensory function in PwD potentially has the additional benefit of providing some relief for their carers or families. Field testing the collection of data required for an economic evaluation in the full RCT was an important aspect of the study. The overall quality of the economic data was good, with minimal missing data for completing dyads, and we have gathered intelligence to guide data collection more precisely. For the RCT, we will use country‐specific utility value sets for the EQ‐5D‐5L and the DEM‐QoL where available at the time of analysis.

Limitations of our field trial include the nonblinded study design and the small sample size. However, undertaking a feasibility study such as this is a necessary step prior to conducting a RCT, and it gave us the opportunity to make iterative changes to the study procedures and invention. We have powered a full RCT of the SI to detect a difference of 4 points on the DEM‐QoL. Assuming that participants in the “care as usual” comparison group do not improve post intervention, a sample size of 354 dyads should detect any clinically important effect of the intervention.

Finally, the strengths of our study include the comprehensiveness of the intervention approach, the international reach, and the variety of methods of ascertaining outcomes, which contributes to the emerging field of sensory support in people living with dementia.

## CONFLICT OF INTEREST

H.A. and S.F. are employed by Starkey Hearing Technologies, and S.Ma. and S.Mo. are employed by Essilor International. There are no other conflicts of interest.

## AUTHOR CONTRIBUTIONS

I.L. is the programme lead and conceptualised, designed and led the field trial. E.H. is the Senior Sensory Support Therapist. Z.S. and A.P.C. are research assistants. R.E., E.C., and H.H. provided health economic input. M.H. provided statistical input for the study. I.H. and L.W. led the qualitative analysis. C.H. and Fo.C. oversaw study delivery in their sites. Fi.C. and E.F. were involved in the study design and interpretation of study results. C.T., H.A., S.F., S.Ma., and S.Mo. provided professional input to the design and conduct of the trial. P.D. is the programme colead. I.L., Z.S., E.H., and W.K.Y. took primary responsibility for writing the paper; all authors were involved in critical revision of the article.

## TRIAL REGISTRATION NUMBER

The trial is a psychosocial intervention with an allocated ISRCTN number 35019114 16 January 2018.

## Supporting information

Chart S1. Flow Chart of Study Procedures
Table S2. Battery of outcome measures
Table S3. Key themes emerging from the semistructured interviews with participant dyads following the basic or extended sensory intervention
Table S4. Nested case series of participants who received the extended intervention: characteristics and outcomes

## Data Availability

The data that support the findings of this study are available from the corresponding author upon reasonable request.
