# Supplementary material for: Impact of an intervention to support hearing and vision in dementia: The SENSE‐Cog Field Trial
Source: Int J Geriatr Psychiatry. 2019 Dec 3;35(4):348–57. doi: 10.1002/gps.5231 (PMC7079053; doi:10.1002/gps.5231)
Supplement: Supplementary file 1 — Chart S1. Flow Chart of Study Procedures Table S2. Battery of outcome measures Table S3. Key themes emerging from the semistructured interviews with participant dyads following the basic or extended sensory intervention Table S4. Nested case series of participants who received the extended intervention: characteristics and outcomes [file GPS-35-348-s001.docx]

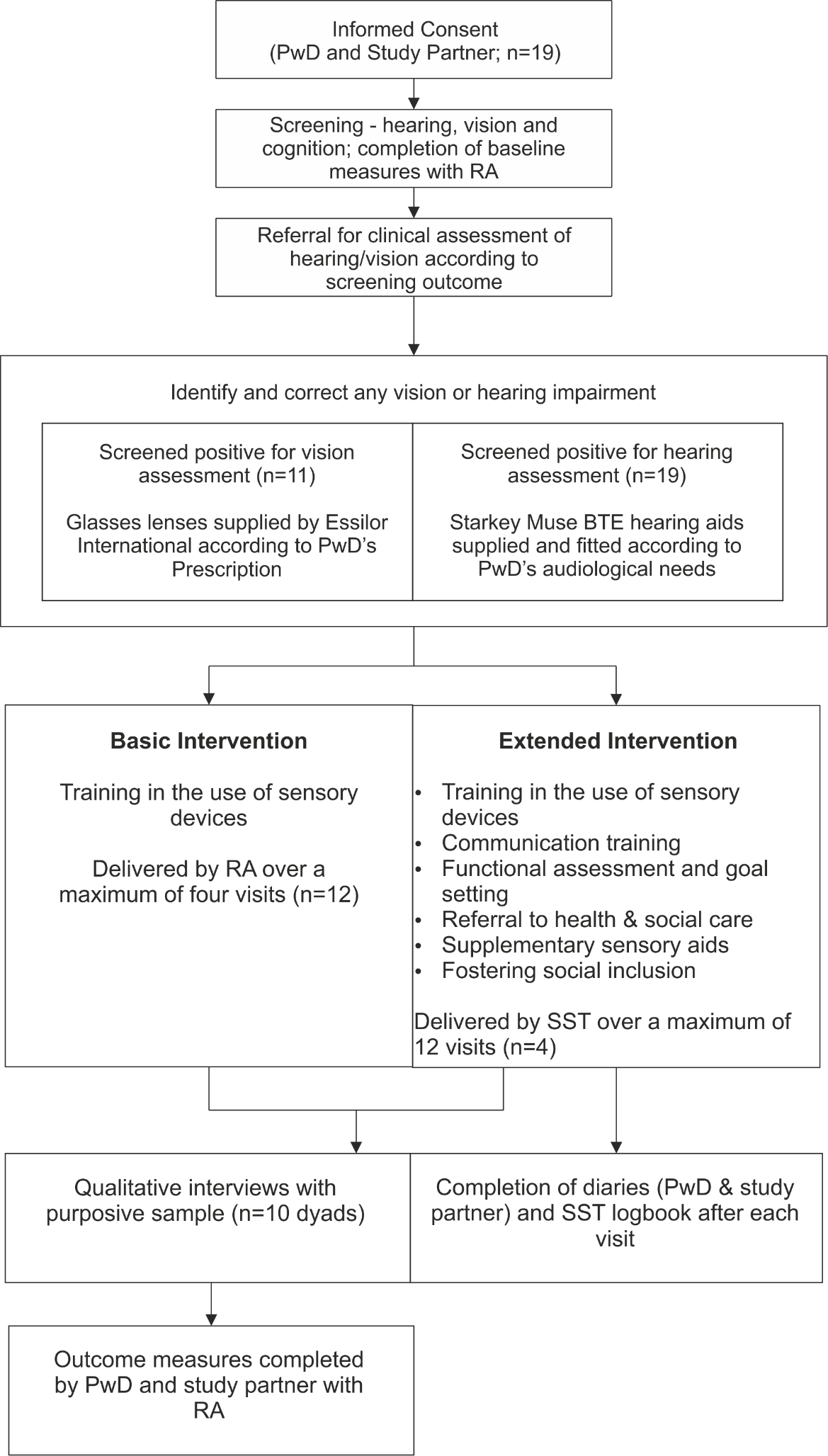
**Supplementary Chart S1: Flow Chart of Study Procedures (adapted from Hooper et al., 2019)^12^**

**Supplementary Table S2: Battery of outcome measures**

| **Outcome domain** | **Tool(s) to capture outcome** |
| --- | --- |
| ***PwD*** | |
| Quality of life | DEM-QoL, Dementia Quality of Life Scale ^16^ and DEM-QoL-Proxy ^25^; SF-12, 12 Item Short Form Survey ^47^ and SF-12 Proxy ^47^; EuroQol-5D-5L, EuroQol 5 Dimensions 5 Levels ^15^ and EuroQol 5D-5L Proxy ^15^ |
| Behaviour | NPI-12, Neuropsychiatric Inventory ^29^ |
| General mental well-being | GSE, Generalised Self-efficacy Scale ^30^ |
| Function | BADLS, Bristol Activities of Daily Living Scale ^32^ |
| ***Study partner*** | |
| Mental health | GDS-15, Geriatric Depression Scale 15 ^34^ |
| Burden and stress | FCRS, Family Caregiving Role Scale ^35^ |
| Health | PHQ-15 ^36^ |
| ***PwD and study partner*** | |
| Relationship | RSS, Relationship Satisfaction Scale ^31^ |
| ***PwD health economic measures*** | |
| Health, social and informal care resource use | Resource Utilisation in Dementia (RUD) Lite ^18^ |
| Health utility | EuroQol-5D-5L (self-rated), EuroQol-5D-5L (proxy-rated), DEM-QoL (self-rated), DEM-QoL(proxy-rated) and associated utility values ^21^ ^22^ |

**Supplementary Table S3: Key themes emerging from the semi-structured interviews with participant dyads following the basic or extended sensory intervention**

| **Theme** | **Exemplar Quote** |
| --- | --- |
| **Improved communication** | **R: and you said that your sons had mentioned an improvement in your communication as well**  SP: yes yeah  P: yeah  **R: would you like to say a bit more about that**  SP: yes my son said that he said ‘oh I've noticed a difference with my dad since he's worn those hearing aids’  P: yeah  SP: he said he's been a lot better yeah and I think you've felt better haven't you PwD?  P: pardon what did you say?  SP: no you've felt better haven't you  P: yeah |
| **PwD’s knowledge and skills regarding the devices** | **R: For sure...Now we move on to some other questions that have to do with usage of your aids and glasses. It is not quite easy to take care of them right?**  SP: it is not that is difficult ... other are the difficulties ...for example the glasses since they are necessary she got used to them and she is ok with them now, she wears them daily the ones for near it is a change and she needs to get used to them and I have to push her so she will wear them. For example once I gave her lentils and I asked her to clean them up so then she was forced to use them or yesterday I gave her to clean some olives and she again did it with the help of the glasses. But her hearing aids no…  SP: and you now take them out yourself and undo the little compartments so that the battery's not enclosed don't you  P: yup, mmmhh  SP: and keep you know you look after them yourself  P: yeah  SP: umm  P: I'll go fetch them  SP: you can do if you want to  P: they're in my bedroom  SP: yeah ok  SP: so you see that’s a huge thing that he now manages to do that himself… |
| **Improved insight** | P: You can say that I have seen benefit, because in the future I will have the (.)  **R: The aids. You mean**  P: the aids.  **R: in case your hearing gets worse?**  P: Yes, in case my hearing starts to get worse, I will realize that something is not ok,  **R: (Uh huh).**  P: if I don't hear well, it means there is an increase of (.)  **R: the loss.**  P: Yes, the loss.  P: it took a long time though  SP: it took a few weeks it took you about 4 weeks didn't it?  P: mmm, mmm, mmmm  SP: and then alot of persuasion and encouragement to wear them  P: Yes… |
| **Advantage of a home-based intervention with therapist support** | **R: Ok. And how did you feel during the visits?**  P: Comfortable (laughs)  **R: Comfortable, comfortable.**  SP: Friendly with everyone.  **R: Friendly with everyone...**  SP: Which is something very important, to feel comfortable and friendly with the person that visits you at home to do the research.  **R: That is right, yes yes,**  SP: Very important  **R: It is very important because indeed you need to feel comfortable in order to be able to ...**  SP: …to express what we want or say something we want, to express it free. I believe it is very important. |
|  | **R: is there anything you would have changed about it?**  SP: only from a selfish point of view, I would have liked to have seen [SST] more afterwards because we enjoyed each other’s company, we used to have good chats and a laugh together  **R: I will pass that on to her**  **R: well, there was lots to cover wasn't there. Did she come about 12 times something like that, since the summer?**  SP: yes, I miss her coming actually  **R: aw, yes I think she enjoyed it**  SP: yes, we did as well. She always had a glass of water (laughs). She didn't like tea or coffee. erm, yes we used to have a good chat together the three of us. |

**Key:** P= PwD; SP= Study partner; R= Researcher; SST=Sensory Support Therapist;

**Supplementary Table S4: Nested case series of participants who received the extended intervention: characteristics and outcomes**

|  | **Case study 1** | **Case study 2** | | **Case study 3** | | **Case study 4** | |  |
| --- | --- | --- | --- | --- | --- | --- | --- | --- |
|  | ***Case Summary:*** | | | | | | |  |
| PwD age; sex | 77 years; female | 74 years; male | | 79 years; male | | 70 years; male | |  |
| Diagnosis, duration since onset of cognitive impairment | Mixed dementia;  18 months | Vascular dementia;  84 months | | Vascular dementia;  60 months | | Alzheimer’s disease;  30 months | |  |
| Study partner type; age | Adult daughter;  54 years | Spouse;  60 years | | Spouse;  76 years | | Spouse;  69 years | |  |
| Hearing impairment^†^ | HearCheck ‘3’ in left and right ear | HearCheck ‘4’ in left and right ear | | HearCheck ‘3’ in left ear; ‘2’ in right ear | | HearCheck ‘4’ in left and right ear | |  |
| Vision impairment^‡^ | No impairment | No impairment | | PEEK score 0.4 | | PEEK score 0.5 | |  |
| Prescription of sensory aids | Bilateral hearing aids | Bilateral hearing aids | | Glasses  Bilateral hearing aids | | Glasses  Bilateral hearing aids | |  |
| ***Description of support system*** | | | | | | | | |
|  | Large and supportive family living close by. Family visit each day and are quite protective of PwD | Supportive wife with a good understanding of dementia. Befriender weekly, Alzheimer’s Society group and contact with a small circle of close friends | | Wife has several medical needs, which limit her function. Two adult sons locally, befriender weekly, dementia group fortnightly, and dementia café fortnightly | | Wife is supportive and protective. Family nearby are in regular contact. Weekly contact with a neighbour for accompanied walks | |  |
| ***Selected baseline variables, PwD:*** | | | | | | | | |
| MoCA | 19/30 | 21/30 | | 12/30 | | 12/30 | |  |
| HHIE-S | 10 | 0^§^ | | 26 | | 8 | |  |
| LV-VFQ-20 | N/A | N/A | | 4.22 | | 1.37 | |  |
| NPI-12 | 4 | 20 | | 6 | | 0 | |  |
| Function (BADLS) | 5 | 9 | | 31 | | 6 | |  |
| DEM-QoL | 91 | 106 | | 87 | | 106 | |  |
| DEM-QoL Proxy | 121 | 111 | | 86 | | 112 | |  |
| ***Components of extended intervention received*** | | | | | | | | |
| Number of visits from the SST | 7 visits over 13 weeks  All intervention components addressed | 8 visits over 11 weeks  All intervention components addressed | | 12 visits over 17 weeks  All intervention components addressed. | | 4 visits over 7 weeks.  3 intervention components addressed in 4 visits before  participant withdrew | |  |
| ***Component 1:*** Assessment, prescription and fitting | One audiology visit for assessment and instant fit | One audiology visit for assessment and instant fit;  Replacement hearing aid required following accidental damage | | Two optometry visits for assessment and fitting;  One audiology visit for assessment and instant fit;  Two further audiology visits required due to functionality problems with the hearing aids | | Two optometry visits for assessment and fitting;  Two audiology visits for assessment and fitting | |  |
| ***Component 2***:  Adherence support | Knowledge and skills support provided | Knowledge and skills support provided | | Knowledge and skills support provided | | Initial information about the devices provided | |  |
| ***Component 3:***  Supplementary aids to enhance function at home | No additional equipment required | No additional equipment required | | Lamp, glasses strap and hearing aid clip were trialled but did not benefit the PwD | | Not addressed due to early withdrawal | |  |
| ***Component 4:***  Communication training | Communication manual enabled discussion about when PwD is disengaging within conversation and ways to manage this | Study partner became more aware of fatigue and saturation in PwD and adjusted expectations accordingly.  Both became more aware of environments that support communication  PwD recognised the benefit of wearing his hearing aids when out in the community | | Communication guidance was completed with study partner to optimise communication through making adjustments | | Not addressed due to early withdrawal | |  |
| ***Component 5:***  Referrals for extra support from health and social care | No referrals required | No referrals required | | Multiple needs were evident and appropriate referrals made, i.e. to GP for review of antidepressant | | Not addressed due to early withdrawal | |  |
| ***Component 6:***  Social inclusion | No intervention required | Signposted to dementia-friendly arts opportunities | | Focused on optimising function during pre-existing social inclusion activities | | Not addressed due to early withdrawal | |  |
| ***Outcomes from the Extended Support Intervention:*** | | | | | | | | |
| 1. **Hearing and vision function:** | | | | | | | | |
| Hearing aids | Positive change in hearing aid skills and knowledge evident on HASK test (+50% knowledge score; +49% skills score) | Positive change in hearing aid skills and knowledge evident on HASK test (+28% knowledge score; +20% skills score) | | Passive recipient due to cognitive and physical limitations; knowledge and skills support directed towards study partner who showed positive change in hearing aid skills and knowledge evident on study partner’s HASK test (+8% knowledge score; +24% skills score) | | No outcome measurement completed due to early withdrawal | |  |
| Glasses | NA | NA | | Sensory support included strategies to position glasses correctly and to locate glasses within the environment. | | No problems with maintenance of glasses. | |  |
| 1. **Adherence with hearing aids and glasses** | | | | | | | | |
|  | Wearing hearing aids for 12 hours a day every day by the end of the SI | PwD’s motivation to use hearing aids increased over the course of the SI, reflected in daily use for approximately 8 hours by the end of the intervention | | PwD had problems with the fit of the hearing aids, which impacted on motivation for wear. Use diminished to wearing the aids for 4 days out of 7 for approximately 4 hours by the end of the SI;  Fully adherent with varifocals | | Wearing hearing aids on 2/3 of days during waking hours;  Fully adherent with varifocals;  Did not use reading glasses | |  |
| 1. **Goal setting and goal attainment:**   Goal performance: 1=cannot do successfully to 10=can do successfully  Goal attainment rated on a 5-point scale (0%, 25%, 50%, 75% or 100%) | | | | | | | | |
| Number of goals set | 3 | | 3 | | 3 | | 2 |  |
| Nature of goals set | Goals 1 & 2: device care;  Goal 3: function at home. | | Goal 1: device use;  Goal 2: communication  Goal 3: social inclusion. | | Goal 1: device use;  Goal 2: communication;  Goal 3: social inclusion. | | Goals 1 & 2: social inclusion. |  |
| Change in mean goal performance rating | +4 (PwD);  +4.7 (study partner) | | +4.2 (PwD);  +4.8 (study partner) | | +2.7 (PwD);  +4.3 (study partner) | | No outcome measurement completed due to early withdrawal |  |
| Goal attainment percentage (SST) | Goal 1 75%  Goal 2 50%  Goal 3 100% | | Goal 1 100%  Goal 2 75%  Goal 3 100% | | Goal 1 100%  Goal 2 75%  Goal 3 75% | |  |  |
| 1. **Participant diary and SST logbook ratings of helpfulness and initiative:**     *Perceived helpfulness by the PwD* - 1: Not at all helpful; 2: Not very helpful; 3: Neutral; 4: Quite helpful; 5: Very helpful  *PwD took the initiative* - 1: strongly disagree; 2: disagree; 3: neutral; 4: agree; 5: strongly agree | | | | | | | | |
|  | Mean score of all ratings; range | | | | | | |  |
| Helpfulness  (PwD diary)  Initiative (Study partner diary)  Initiative  (SST logbook) | 5.00  4.29 (4-5)  3.83 (3-4) | 4.67 (4-5)  4.22 (3-5)  4.77 (4-5) | | 3.91 (3-5)  2.36 (2-3)  2.91 (2-4) | | 3.75 (3-4)  3.50 (3-4)  4.50 (4-5) | |  |
| 1. **Qualitative findings from participant diaries and SST logbooks** | | | | | | | | |

| **Impact of intervention on key outcomes:** | | | | |
| --- | --- | --- | --- | --- |
| **Hearing function** | Following provision of aids, TV volume reduced by half.  Study partner reported improved communication, able to follow multiple strands of conversation and overall more engaged with family. | Study partner reported PwD’s functional ability improved, was more alert and more engagement with family life, and doing more activities when wearing aids; “I feel like I’ve got him back”;    PwD reported feeling more confident and “vocally alive”. | Study partner reported she no longer had to shout or repeat herself;  Interactions between the dyad were calmer and relationship quality improved;  PwD was more actively engaged during the dementia group. | Study partner reported that she did not have to repeat herself as much, which improved relationship quality. |
| **Vision function** | NA | NA | No major changes reported in visual function.  Contrast was used successfully to aid PwD’s ability to locate glasses. | PwD reported that varifocals were “fantastic”.  Sunglasses helped with managing glare outdoors.  PwD continued to have difficulty reading and did not find near vision glasses beneficial. |
| **Overall impact on PwD reported by study partner** | “Mum was pleased with the knowledge retained on the use of hearing aids” | “[PwD] is clearly benefitting from his aids-talking himself about the impact they have made” | “Improvement in hearing immediately evident” | N/A |
| **Feedback on aspects of the intervention reported by study partner or SST** | | | | |
| Engagement of the PwD with the intervention | “All went well, [PwD] joined in the discussions and added her own comments in” ^SP^ | “[PwD] is much more confident asking questions about his [hearing] aids.” ^SP^ | “[PwD] seems to be (in) more control and interested.” ^SP^ | “[PwD] was interested and motivated.” ^SP^ |
| Meaningfulness of intervention in daily life | “Goals agreed, will help [PwD] in her everyday activities.” ^SP^ | “Social inclusion signposting was well received- [PwD] expressed positive anticipation of these opportunities.” ^SST^  “Developing ideas for future hospital admissions to maintain effective communication. Planning to meld ‘This is me’ with ‘hearing hygiene’!” ^SP^ | “Discussed ways of helping [PwD] with finding his specs” ^SP^ | “[PwD] realised that he would benefit from wearing his hearing aids all the time.” ^SP^ |
| Positive feedback on intervention | “I think the level of info was enough so as not to overpower [PwD] also gave food for thought on future meetings.” ^SP^  “Goal-setting led nicely from hearing aid questionnaire […] and was straightforward to complete. ^SST^ | “The communications info was great. Very encouraging.” ^SP^ | “The audiologist adjusted his approach to suit the needs of the participant. Hearing aids’ fitting was completed.” ^SST^ | “The glasses and hearing aid use questionnaires elicited open discussion about use of the aids and factors underpinning this” ^SST^ |
| Negative feedback on intervention | “If there was a list of things to be discussed at each visit then we could make closer observations prior.” ^SP^ | “[PwD] presented as fatigued by the end of the visit.” ^SST^  “I [SST] provided written information about the product […]. This contained extraneous information about other aids & types of fitting. Tailored information specific to aid would be better.” ^SST^ | “[PwD] seemed to lose interest.” ^SP^  “Problems with fit impact upon confidence and willingness to use [hearing] aids” ^SST^ | “Reduce session to ½ hour” ^SP^  “There are too many questions. [PwD] becomes very tired and if he does not know the answer he feels he has failed.” ^SP^ |

*Abbreviations: MoCA, Montreal Cognitive Assessment*^39^*; HHIE-S, Hearing Handicap Inventory for the Elderly Screening tool; LVVFQ-20, Low Vision Visual Functioning Questionnaire-20; NPI-12, Neuropsychiatric Inventory 12; BADLS, Bristol Activities of Daily Living Scale; Dem-QOL(-P), dementia quality of life (-Proxy); SST, Sensory Support Therapist; PwD, person with dementia;*

^†^*The HearCheck screener provides a count of detected signals at or above threshold levels for 2 frequencies (3 levels per frequency) and gives the number of tones detected from 0 – 6 for each ear* ^40^*.*

^‡^*‘Mild’ (+ 0.2 to 0.5 LogMAR) visual impairment measured by the PEEK screening tool* ^41^ *according to ICD-10 (2016) classification* ^43^

^§^*This participant lacked awareness of his hearing deficit at baseline, thus rated himself as ‘0’ on the HHIE-S; in spite of this he benefitted from the intervention.*
